# Stroke of antiplatelet and anticoagulant therapy in patients with coronary artery disease: a meta-analysis of randomized controlled trials

**DOI:** 10.1186/s12872-021-02384-w

**Published:** 2021-12-01

**Authors:** Qiao Yu Shao, Zhi Jian Wang, Xiao Teng Ma, Xu Ze Lin, Liu Pan, Yu Jie Zhou

**Affiliations:** 1 Anzhen Hospital, Capital Medical University, Beijing Institute of Heart Lung and Blood Vessel Disease, The Key Laboratory of Remodeling-Related Cardiovascular Disease, Ministry of Education, Beijing, China; 2 Department of Cardiology, Beijing Anzhen Hospital, Capital Medical University, Anzhen Avenue #2, Chaoyang District, Beijing, 100029 China

**Keywords:** Coronary artery disease, Stroke, Antiplatelet, Anticoagulant, Meta-analysis

## Abstract

**Background:**

We performed a meta-analysis sought to investigate the risk of stroke with antiplatelet and anticoagulant therapies among patients with coronary artery disease (CAD).

**Methods:**

We searched PubMed, EMBASE, and Cochrane Library for randomized controlled trials from January 1995 to March 2020. Studies were retrieved if they reported data of stroke for patients with CAD and were randomized to receive intensive versus conservative antithrombotic therapies, including antiplatelet and oral anticoagulant (OAC). Analyses were pooled by random-effects modeling. A total of 42 studies with 301,547subjects were enrolled in this analysis.

**Results:**

Intensive antithrombotic therapy significantly reduced risk of all stroke (RR 0.86, 95% CI 0.80–0.94) and ischemic stroke (RR 0.80, 95% CI 0.71–0.91), but increased risk of hemorrhagic stroke (RR 1.36, 95% CI 1.00–1.86) and intracranial hemorrhage (RR 1.46, 95% CI 1.17–1.81). Subgroup analyses indicated that OAC yields more benefit to all stroke than antiplatelet therapy (OAC: RR 0.73, 95% CI 0.58–0.92; Antiplatelet: RR 0.90, 95% CI 0.83–0.97; Between-group heterogeneity *P* value = 0.030). The benefit of antiplatelet therapy on all stroke and ischemic stroke were mainly driven by the studies comparing longer versus shorter duration of dual antiplatelet therapy (All stroke: RR 0.86, 95% CI 0.78–0.95; ischemic stroke: RR 0.84, 95% CI 0.75–0.94).

**Conclusions:**

Among CAD patients who have already received antiplatelet therapy, either strengthening antiplatelet or anticoagulant treatments significantly reduced all stroke, mainly due to the reduction of ischemic stroke, although it increased the risk of hemorrhagic stroke and intracranial hemorrhage. OAC yields more benefit to all stroke than antiplatelet therapy.

**Supplementary Information:**

The online version contains supplementary material available at 10.1186/s12872-021-02384-w.

## Background

Stroke is a devastating clinical event associated with substantial mortality and morbidity [1]. Patient with coronary artery disease (CAD) always have a high prevalence of stroke due to concomitant atherosclerotic disease of the cerebral vascular system or cardiogenic embolism [2, 3]. Nevertheless, the pathophysiology and causes of stroke are more diverse than those in ischemic coronary syndromes. Either ischemic or hemorrhagic stroke can cause more deterioration in the quality of life compared with other ischemic or bleeding events, even if patients who survive in the acute period [4]. Any type of stroke is thought to result in a life-long reduction in utility and have a much greater impact on the quality of life, regardless of the severity of stroke [5–9].

Antiplatelet and anticoagulant treatments play pivotal roles throughout the prevention of cardiovascular and cerebrovascular disease. Dual antiplatelet therapy (DAPT) has been recommended for patients with acute coronary syndromes (ACS) and those undergoing percutaneous coronary intervention given its benefit in the risk of stent-related and spontaneous recurrent ischemic events [10, 11]. However, considering the improved safety and efficacy of drug-eluting stents (DES) [12, 13] and advances in medical treatment [14–16], the optimal duration of DAPT in patients with ACS remains controversial. While more powerful antithrombotic strategy might be beneficial to reduction of ischemic stroke (IS), it leads to a higher risk of hemorrhagic stroke (HS) or intracranial hemorrhage (ICH). Compared with spontaneous HS, oral anticoagulant (OAC) related intracerebral hemorrhage has a larger hematoma volume [17] and a worse prognosis [18–20]. Therefore, it is essential to figure out the safety and efficacy of intensive antithrombotic therapy (ATT) for stroke of CAD populations. Additionally, clinical evidence supported antiplatelet for non-cardioembolic stroke prevention, while anticoagulant is more recommended for the prevention of most types of cardioembolic stroke [21]. However, whether antiplatelet therapy and OAC yield the same benefit in the risk of stroke among CAD population is not clear.

Therefore, we conducted a systematic review and meta-analysis to investigate whether escalation of ATT, including antiplatelet therapy and OAC, is beneficial in different types of the stroke among patients with CAD.

## Method

### Study design

Eligible studies for this meta-analysis were randomized controlled trials (RCT) of patients with CAD treated with OAC or antiplatelet therapy and provided at least 1 endpoint of any type of stroke. Studies were included if they compared the treatment effects of intensive versus conservative ATT, specifically including long-term versus short-term, novel P_2_Y_12_ inhibitor (ticagrelor or prasugrel) versus clopidogrel, combined (DAPT or OAC combine antiplatelet therapy) versus alone, (all above are in the order of intensive therapy versus conservative therapy). Besides, the studies were excluded if they met any of the following criteria: (1) Individualized ATT based on platelet function or genetic monitoring; (2) Patients with atrial fibrillation (AF) or other diseases that need to receive long-term OAC treatment; (3) The total number of participants was less than 1000 or follow-up duration was shorter than 6 months; (4) Phase I and phase II clinical trials; (5) Patients were not randomly assigned at the time of receiving ATT.

### Search strategy

We retrieved RCT through PubMed, EMBASE, and Cochrane Library using the keywords relating to ATT (“platelet aggregation inhibitors”, “anticoagulants”, “antithrombotic”, “NOAC”, “clopidogrel”, “aspirin”, “thienopyridine”) and CAD (“acute coronary syndrome”, “percutaneous coronary intervention”). Detailed search strategies are demonstrated in Additional file 1. To minimize heterogeneity due to rapidly advancing treatment strategies, we only included studies published from 1 January 1995 to 12 March 2020. Only articles written or published in English were included.

### Trial selection and data extraction

**Fig. 1:**
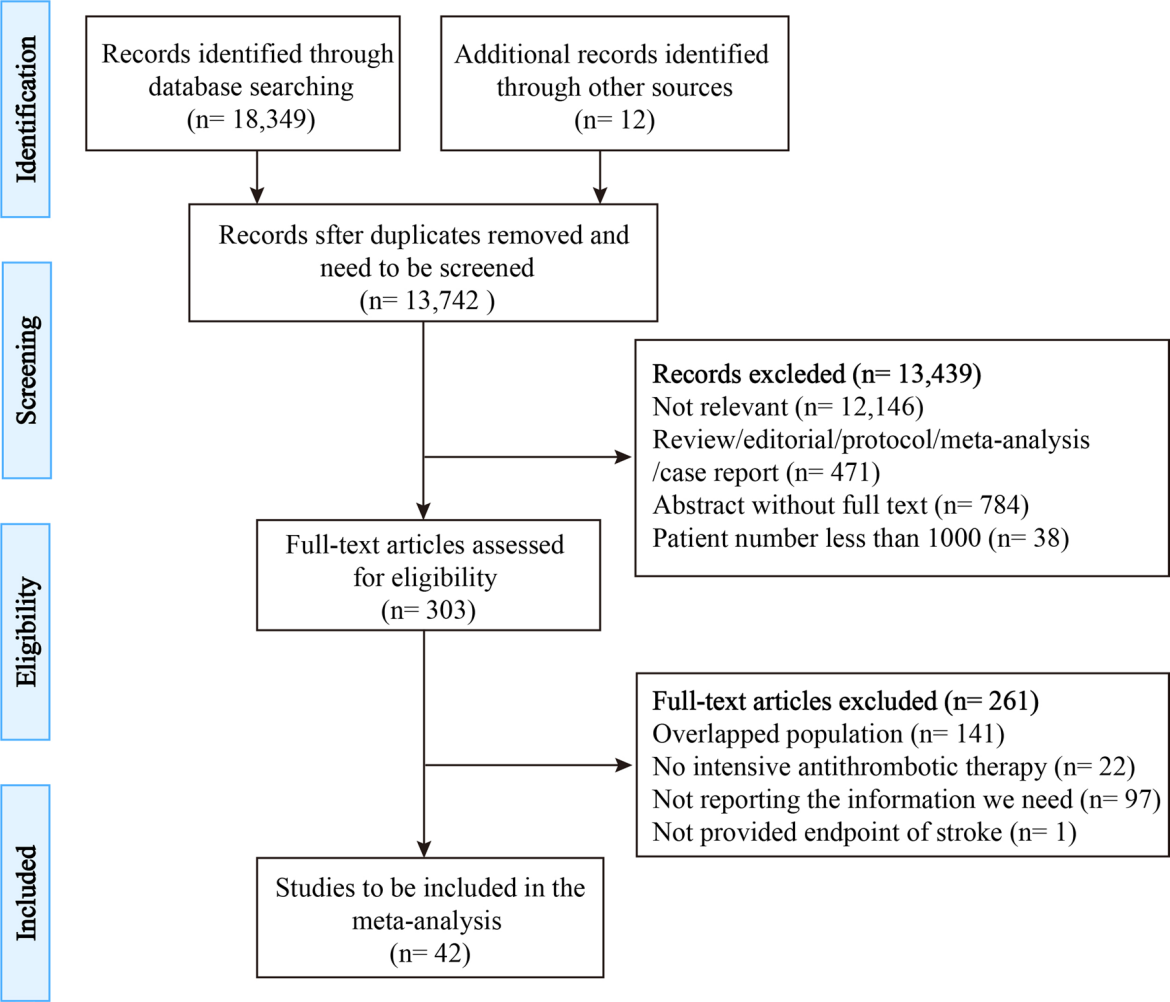
Flow chart of study selection

Two investigators (Q.Y.S. and X.T.M.) independently screened the titles, abstracts, and full text to authenticate whether they met the inclusion criteria, and categorized ATT to OAC or antiplatelet therapy among each trial as recommended in the guidelines [10]. Antiplatelet therapies were also subdivided into long-term versus short-term DAPT (also including DAPT vs. monotherapy), novel P_2_Y_12_ inhibitor versus clopidogrel, and others (Orbofiban, Cilostazol, Vorapaxar) versus placebo. Data recorded included first author, journal, year of publication, study name, study population, baseline clinical characteristics, interventions, and outcomes of all types of the stroke. The authors were contacted via email when the data remained unclear or needed access to additional data. The filtering process is shown in the flowchart (Fig. 1). If there were several articles from the same group of subjects, we chose the one with the longest follow-up data. Conflicts between investigators were resolved by consensus and consulting a third investigator (Z.J.W.). The methodological quality of RCT was assessed by Cochrane’s Collaboration tool for evaluating the risk of bias (Additional file 1: Table S1).

### Endpoints and definitions

The primary endpoint of interest was all stroke. We also extracted and analyzed IS, HS, and ICH. We excluded those studies in which the study population overlapped completely or partially unless the original study could be clearly abstract information from different populations and endpoints. The principal analyses were performed in the intention-to-treat populations.

### Statistical analysis

Individual study’s baseline characteristics, risk estimates, and raw outcome data were extracted from each RCT. Data for all endpoints were pooled and analyzed using DerSimonian and Laird random-effects models [22]. The percentage of variability across studies caused by heterogeneity beyond chance was evaluated with the Cochrane test and calculated with I^2^ statistic. Values < 25% indicating low, 25–50% indicating moderate, and > 50% indicating high heterogeneity [23]. Given the potential difference in the treatment effects between antiplatelet therapy and OAC, we planned pre-specified subgroup analysis according to types of ATT for all the endpoints. *P* values for between-group heterogeneity were all from meta-regression. Meta-regression analysis was performed to explore pre-defined sources of heterogeneity of stroke. The pre-defined covariates included study sample size, follow-up time, mean age, the proportion of women, smokers, and diabetes. Sensitivity analyses were examined by excluding one study at a time. Publication bias was assessed by Egger’s linear regression test [24], Begg’s test, and visual inspection of funnel plots. If the results between bias tools are different, we used the trim-and-fill method to further evaluate and adjust publication bias. Statistical analysis was performed using Stata 12.0 (Stata Corp). The results were regarded as statistically significant at 2-tailed *P* < 0.05.

## Results

A total of 8259 articles were retrieved after duplication removal, of which 264 articles warranted full-text review for detail. We finally identified 42 studies (301,547 enrolled patients) that met the inclusion criteria and provided at least 1 endpoint of interest (Fig. 1). Among 42 RCT involved patients referred for CAD, 15 studies (153,856 enrolled patients) were randomized after diagnosis of ACS, which combined unstable angina, Non-ST-elevation myocardial infarction, and ST-elevation myocardial infarction; 2 studies (37,498 enrolled patients) were about stable CAD; 23 studies (89,569 enrolled patients) were patients with ACS or stable CAD undergoing percutaneous coronary intervention, including 18 with DES, 1 with bare-metal stent [25], 3 with DES or bare-metal stent [26–28], and 1 was unreported [29]. The remaining 2 studies incorporated 1 specifically for CAD accompany heart failure (HF) [30], 1 for cardiovascular disease or multiple risk factors [31]. One study enrolled only veterans [32]. The quality assessment and characteristics of included studies are presented in Additional file 1: Tables S1 and S2.

**Fig. 2:**
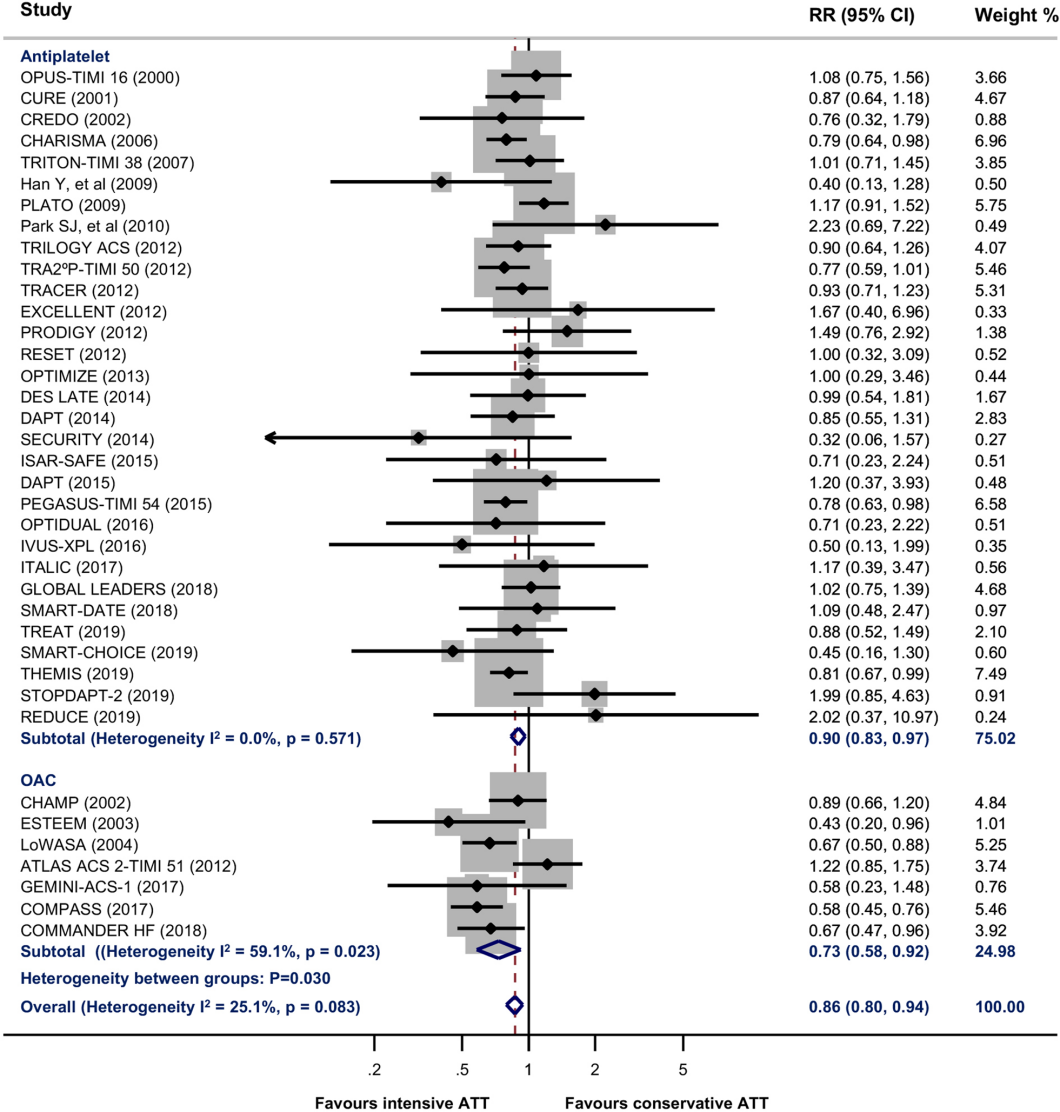
Estimates of risk for all stroke between intensive antithrombotic therapy and conservative antithrombotic therapy

**Fig. 3:**
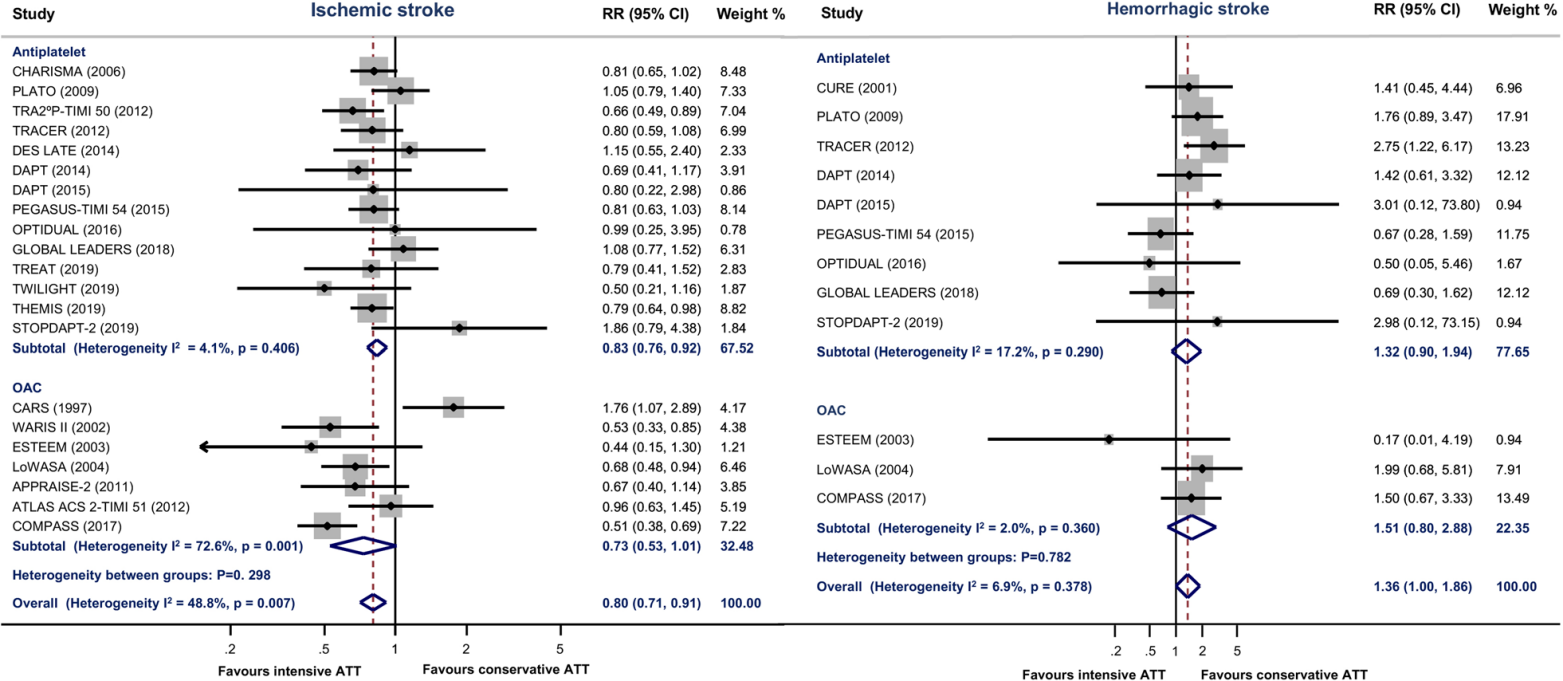
Estimates of risk for ischemic stroke and hemorrhagic stroke between intensive antithrombotic therapy and conservative antithrombotic therapy

Compared with conservative ATT, intensive ATT was associated with a significantly lower risk of all stroke (RR 0.86, 95% CI 0.80–0.94; *P* = 0.001) (Fig. 2). There was a high between-study heterogeneity within OAC group (I^2^ = 59.1%, *P* = 0.023), but no heterogeneity within antiplatelet group (I^2^ = 0.0%, *P* = 0.571). Intensive ATT also reduced the risk of IS (RR 0.80, 95% CI 0.71–0.91; *P* = 0.001) (Fig. 3), but increased the risk of HS (RR 1.36, 95% CI 1.00–1.86; *P* = 0.051) (Fig. 3) and ICH (RR 1.46, 95% CI 1.17–1.81, *P* = 0.001) (Additional file 1: Fig. S1). Subgroup analyses found that OAC yielded more benefit in all stroke than antiplatelet therapy (OAC: RR 0.73, 95% CI 0.58–0.92, *P* = 0.006; Antiplatelet therapy: RR 0.90, 95% CI 0.83–0.97, *P* = 0.004; *P* value for between-group heterogeneity = 0.030). There was no significant difference in the treatment effect of IS, HS, or ICH between antiplatelet and OAC subgroups (Table 1).

**Table 1:** Subgroup analyses of antiplatelet and anticoagulant treatments for the stroke outcomes

Variable	No. of studies	Estimates and 95% CI	*P* value	I^2^ (%)	*P* value for within-group heterogeneity	*P* value for between-group heterogeneity^a^
All stroke	39	0.86 (0.80, 0.94)	0.001	25.1	0.083	0.030
OAC	7	0.73 (0.58, 0.92)	0.006	59.1	0.023	
Antiplatelet	32	0.90 (0.83, 0.97)	0.004	0.0	0.571	
Long-term versus short-term DAPT	24	0.86 (0.78, 0.95)	0.002	0.0	0.672	
Novel P_2_Y_12_ inhibitor	4	1.03 (0.87, 1.22)	0.701	0.0	0.584	
Others^b^	4	0.88 (0.71, 1.08)	0.219	25.3	0.260	
Ischemic stroke	21	0.80 (0.71, 0.91)	0.001	48.8	0.007	0.298
OAC	7	0.73 (0.53, 1.01)	0.059	72.6	0.001	
Antiplatelet	14	0.83 (0.76, 0.92)	< 0.001	4.1	0.406	
Long-term versus short-term DAPT	10	0.84 (0.75, 0.94)	0.002	0.0	0.479	
Novel P_2_Y_12_ inhibitor	2	1.00 (0.77, 1.30)	0.981	0.0	0.432	
Others^b^	2	0.72 (0.58, 0.90)	0.003	0.0	0.388	
Hemorrhagic stroke	12	1.36 (1.00, 1.86)	0.051	6.9	0.378	0.782
OAC	3	1.51 (0.80, 2.88)	0.204	2.0	0.360	
Antiplatelet	9	1.32 (0.90, 1.94)	0.160	17.2	0.290	
Long-term versus short-term DAPT	7	0.96 (0.62, 1.49)	0.858	0.0	0.709	
Novel P_2_Y_12_ inhibitor	1	1.76 (0.89, 3.47)	0.103	NA	NA	
Others^b^	1	2.75 (1.22, 6.17)	0.014	NA	NA	
Intracranial hemorrhage	19	1.46 (1.17, 1.81)	0.001	37.7	0.050	0.465
OAC	5	2.01 (1.00, 4.02)	0.049	63.4	0.027	
Antiplatelet	14	1.39 (1.12, 1.74)	0.003	27.8	0.157	
Long-term versus short-term DAPT	7	1.37 (1.08, 1.75)	0.011	0.0	0.654	
Novel P_2_Y_12_ inhibitor	4	1.15 (0.78, 1.71)	0.475	18.4	0.298	
Others^b^	3	1.64 (0.77, 3.47)	0.197	73.4	0.023	

All estimates and *P* values were analyzed using the random effects model

*OAC* oral anticoagulant

^a^*P* value for between-group heterogeneity refers to the heterogeneity between OAC and Antiplatelet groups

^b^Others subgroup including 1 study for Orbofiban, 1 study for Cilostazol, 2 study for Vorapaxar

Subgroup analyses showed that the effects of antiplatelet therapy were mainly driven by long-term DAPT. Long-term DAPT was of obvious benefit on all stroke (RR 0.86, 95% CI 0.78–0.95; *P* = 0.002) and IS (RR 0.84, 95% CI 0.75–0.94; *P* = 0.002), but had no advantage over HS (RR 0.96, 95% CI 0.62–1.49; *P* = 0.858) and even increased the risk of ICH (RR 1.37, 95% CI 1.08–1.75; *P* = 0.011) compared with short-term DAPT (Table 1). Novel P_2_Y_12_ inhibitor did not show a significant benefit to any type of stroke. Pre-defined subgroup analysis based on the type of CAD showed that intensive ATT significantly reduced all stroke in both ACS (RR 0.89, 95% CI 0.79–0.99; *P* = 0.033) and non-ACS (RR 0.73, 95% CI 0.63–0.85; *P* < 0.001) populations, and there is no heterogeneity between two groups (*P* value for between-group heterogeneity = 0.066) (Table 2). Meta-regression found no study-level covariates which explained the variability of all stroke, IS or HS. No apparent systematic bias was found, and no individual study unduly influenced the effects estimates in the sensitivity analyses.

**Table 2:** Subgroup analyses of ACS and non-ACS population for the stroke outcomes

Variable	No. of studies	Estimates and 95% CI	*P* value	I^2^ (%)	*P* value for within-group heterogeneity	*P* value for between-group heterogeneity^a^
All stroke	20	0.84 (0.76, 0.92)	< 0.001	41.7	0.027	0.066
ACS	16	0.89 (0.79, 0.99)	0.033	32.3	0.104	
Non-ACS	4	0.73 (0.63, 0.85)	< 0.001	35.0	0.202	
Ischemic stroke	14	0.78 (0.67, 0.89)	< 0.001	55.8	0.006	0.440
ACS	11	0.81 (0.68, 0.96)	0.015	52.3	0.021	
Non-ACS	3	0.70 (0.54, 0.91)	0.008	71.5	0.030	
Hemorrhagic stroke	7	1.51 (1.00, 2.27)	0.048	22.8	0.255	0.999
ACS	6	1.49 (0.90, 2.48)	0.123	35.6	0.170	
Non-ACS	1	1.50 (0.67, 3.33)	0.324	–	–	
Intracranial hemorrhage	15	1.41 (1.11, 1.79)	0.005	48.1	0.019	0.517
ACS	12	1.51 (1.10, 2.09)	0.012	54.9	0.011	
Non-ACS	3	1.27 (0.98, 1.66)	0.076	0.60	0.366	

^a^*P* value for between-group heterogeneity refers to the heterogeneity between OAC and Antiplatelet groups

## Discussion

We presented a meta-analysis of all published RCT evaluating the stroke outcomes of intensive versus conservative ATT involving 301,547 CAD patients, with an average follow-up of 20.6 months. We found that among patients with CAD who have already received antiplatelet therapy, intensive ATT, either escalation of antiplatelet therapy or addition of OAC, significantly reduced the risk of all stroke and IS, but increased the risk of HS and ICH compared with conservative ATT. OAC was more effective than antiplatelet therapy in the prevention of all stroke.

Stroke has a profound impact on mortality and morbidity given its high risk of death and irreversible sequelae which explicitly decrease the quality of life. Furthermore, brain–heart interactions leading to post-stroke cardiac injury called “stroke-heart syndrome” (SHS) including acute MI, HF, AF, and sudden cardiac death [33]. Therefore, while it is a rare event, small absolute differences in stroke are clinically significant. In this analysis, we found that among CAD patients who have already received antiplatelet therapy, the escalation of either OAC or antiplatelet therapy significantly reduced the risk of IS, but was accompanied by an increase of HS and ICH. It is not surprising that more intensive ATT results in a lower risk of ischemia and a higher risk of bleeding.

Although it has been well established that OAC is more effective than antiplatelet therapy in the prevention of stroke among patients with AF [34, 35], the relative benefit of OAC versus antiplatelet in stroke among CAD patients is not yet clear. In a Cochrane review analysis, there was no difference between vitamin K antagonists versus antiplatelet therapy in the outcome of any recurrent stroke among patients with presumed arterial origin [36]. Similarly, in the two recent trials, neither rivaroxaban nor dabigatran was found to be superior to aspirin in preventing recurrent stroke after embolic stroke of undermined source [37, 38]. In our study, we found that among CAD patients who have already received antiplatelet therapy, although both OAC and enhanced antiplatelet therapy significantly reduced the risk of all stroke, OAC reduced an extra 17% occurrence of stroke events compared with antiplatelet therapy. The reason for this difference between OAC and antiplatelet therapy is not known. It has been well-established that OAC effectively prevents ‘red’ fibrin clots in areas of reduced or stagnant blood flow, such as the fibrillating left atrium, whereas antiplatelet drugs are effective in preventing ‘white’ platelet clots in areas of high shear stress, such as arterial atherosclerosis thromboembolism [39]. According to the constituent ratio of TOAST classification of stroke, cardioembolic stroke accounted for 21% which is more than large-artery atherosclerosis stroke accounted for 18% [40]. Although we excluded studies that exclusively enrolled patients with AF or other diseases who need to receive long-term OAC treatment, not all the studies completely excluded patients with AF. Golwala et al. [41] claimed in meta-analysis that dual (DATT) and triple antithrombotic therapy (TATT) are equivalent in preventing cardiovascular events with DATT being safer by approximately halving bleeding risk. While Gragnano et al. [42] did not concur with the conclusive statement since the heterogeneity between the duration of TATT in AF population. Therefore, whether the variety in the proportion of patients with AF as well as stroke sources can explain our finding warrants further study.

With the introduction and widespread adoption in the clinical practice of novel P_2_Y_12_ inhibitors, it has been speculated that clopidogrel may yield less additional inhibition of platelet aggregation and clinical benefit compared with more potent novel P_2_Y_12_ receptor inhibitors. In a post-hoc analysis of Assessment of Dual Antiplatelet Therapy with Drug-Eluting Stents (ADAPT-DES) trial, high platelet reactivity, indicated by clopidogrel responsiveness, was independently predicted increased risk for IS [43]. The magnitude of increase in the risk of IS was greater per lesser degrees of residual P_2_Y_12_ receptor inhibition, which implies that more potent inhibitors of platelet aggregation and activation would reduce the frequency of stroke. However, this assumption has not been supported by clinical evidence. In our subgroup analysis, neither prasugrel nor ticagrelor was found superior to clopidogrel for preventing any type of stroke. Therefore, further research is warranted to determine the optimal antiplatelet regimen for the prevention of stroke in patients with CAD. Additionally, subgroup analysis also demonstrated that the efficacy of antiplatelet therapy was mainly driven by long-term DAPT subgroup, which reduced all stroke by 14% and reduced IS by 16%. And we failed to find significant heterogeneity between ACS and non-ACS patients in any endpoint.

### Study limitations

There are certain limitations to this study. First, pooling data of our meta-analysis were based on heterogeneous patient cohorts, designs, as well as diagnostic modalities, although pre-defined subgroup analyses and meta-regression were performed to explore the source of heterogeneity, these results of ATT escalation just explain study-level values but not individual patients. Second, the definition of intensive ATT varied among trials, including long-term DAPT versus short-term DAPT, novel P_2_Y_12_ inhibitor versus clopidogrel, etc. However, although the included antiplatelet regimens and participants were mixed, the heterogeneity of antiplatelet was 0%, and the total heterogeneity was also not significant, indicating that diverse regiments did not lead to significant heterogeneity.

## Conclusion

In conclusion, among CAD patients who have already received antiplatelet therapy, either enhanced antiplatelet or anticoagulant treatments significantly reduced all stroke. The therapeutic effect of OAC for all stroke was more obvious than antiplatelet. Whether this extra benefit of OAC versus antiplatelet therapy is consistent between patients with and without AF warrants further study.

## Supplementary Information

**Additional file 1**. Detailed search strategies;** Tables S1**. Cochrane risk of bias for the individual studies included; Table S2. Characteristics of included studies;** Figure S1**. Estimates of risk for intracranial hemorrhage between intensive antithrombotic therapy and conservative antithrombotic therapy;** Figure S2**. Estimates of risk for all stroke between intensive antithrombotic therapy and conservative antithrombotic therapy for subgroup of antiplatelet;** Figure S3**. Estimates of risk for ischemic stroke between intensive antithrombotic therapy and conservative antithrombotic therapy for subgroup of antiplatelet;** Figure S4**. Estimates of risk for hemorrhagic stroke between intensive antithrombotic therapy and conservative antithrombotic therapy for subgroup of antiplatelet;** Figure S5**. Estimates of risk for intracranial hemorrhage between intensive antithrombotic therapy and conservative antithrombotic therapy for subgroup of antiplatelet;** Figure S6**. Estimates of risk for all stroke between intensive antithrombotic therapy and conservative antithrombotic therapy for subgroup of ACS;** Figure S7**. Estimates of risk for ischemic stroke between intensive antithrombotic therapy and conservative antithrombotic therapy for subgroup of ACS;** Figure S8**. Estimates of risk for hemorrhagic stroke between intensive antithrombotic therapy and conservative antithrombotic therapy for subgroup of ACS;** Figure S9**. Estimates of risk for intracranial hemorrhage between intensive antithrombotic therapy and conservative antithrombotic therapy for subgroup of ACS.

## Data Availability

The datasets used and/or analyzed during the current study are available from the corresponding author on reasonable request.

## Abbreviations

ACS: Acute coronary syndromes
AF: Atrial fibrillation
ATT: Antithrombotic therapy
CAD: Coronary artery disease
DAPT: Dual antiplatelet therapy
DES: Drug-eluting stents
HS: Hemorrhagic stroke
IS: Ischemic stroke
ICH: Intracranial hemorrhage
OAC: Oral anticoagulant
RCT: Randomized controlled trials
