# Supplementary material for: Stroke of antiplatelet and anticoagulant therapy in patients with coronary artery disease: a meta-analysis of randomized controlled trials
Source: BMC Cardiovasc Disord. 2021 Dec 1;21:574. doi: 10.1186/s12872-021-02384-w (PMC8638430; doi:10.1186/s12872-021-02384-w)
Supplement: Supplementary file 1 — Additional file 1. Detailed search strategies; Tables S1. Cochrane risk of bias for the individual studies included; Table S2. Characteristics of included studies; Figure S1. Estimates of risk for intracranial hemorrhage between intensive antithrombotic therapy and conservative antithrombotic therapy; Figure S2. Estimates of risk for all stroke between intensive antithrombotic therapy and conservative antithrombotic therapy for subgroup of antiplatelet; Figure S3. Estimates of risk for ischemic stroke between intensive antithrombotic therapy and conservative antithrombotic therapy for subgroup of antiplatelet; Figure S4. Estimates of risk for hemorrhagic stroke between intensive antithrombotic therapy and conservative antithrombotic therapy for subgroup of antiplatelet; Figure S5. Estimates of risk for intracranial hemorrhage between intensive antithrombotic therapy and conservative antithrombotic therapy for subgroup of antiplatelet; Figure S6. Estimates of risk for all stroke between intensive antithrombotic therapy and conservative antithrombotic therapy for subgroup of ACS; Figure S7. Estimates of risk for ischemic stroke between intensive antithrombotic therapy and conservative antithrombotic therapy for subgroup of ACS; Figure S8. Estimates of risk for hemorrhagic stroke between intensive antithrombotic therapy and conservative antithrombotic therapy for subgroup of ACS; Figure S9. Estimates of risk for intracranial hemorrhage between intensive antithrombotic therapy and conservative antithrombotic therapy for subgroup of ACS. [file 12872_2021_2384_MOESM1_ESM.docx]

**Stroke of antiplatelet and anticoagulant therapy in patients with coronary artery disease: a meta-analysis of randomized controlled trials.**

**Additional File 1**

Table and Figure contents

[Detailed search strategies 2](#_Toc60394307)

[Table S1. Cochrane risk of bias for the individual studies included. 3](#_Toc60394308)

[Table S2. Characteristics of included studies 5](#_Toc60394309)

[Figure S1. Estimates of risk for intracranial hemorrhage between intensive antithrombotic therapy and conservative antithrombotic therapy. 9](#_Toc60394310)

[Figure S2. Estimates of risk for all stroke between intensive antithrombotic therapy and conservative antithrombotic therapy for subgroup of antiplatelet. 10](#_Toc60394311)

[Figure S3. Estimates of risk for ischemic stroke between intensive antithrombotic therapy and conservative antithrombotic therapy for subgroup of antiplatelet. 11](#_Toc60394312)

[Figure S4. Estimates of risk for hemorrhagic stroke between intensive antithrombotic therapy and conservative antithrombotic therapy for subgroup of antiplatelet. 12](#_Toc60394313)

[Figure S5. Estimates of risk for intracranial hemorrhage between intensive antithrombotic therapy and conservative antithrombotic therapy for subgroup of antiplatelet. 13](#_Toc60394314)

[Figure S6. Estimates of risk for all stroke between intensive antithrombotic therapy and conservative antithrombotic therapy for subgroup of ACS. 14](#_Toc60394315)

[Figure S7. Estimates of risk for ischemic stroke between intensive antithrombotic therapy and conservative antithrombotic therapy for subgroup of ACS. 15](#_Toc60394316)

[Figure S8. Estimates of risk for hemorrhagic stroke between intensive antithrombotic therapy and conservative antithrombotic therapy for subgroup of ACS. 16](#_Toc60394317)

[Figure S9. Estimates of risk for intracranial hemorrhage between intensive antithrombotic therapy and conservative antithrombotic therapy for subgroup of ACS. 17](#_Toc60394318)

[Reference 18](#_Toc60394319)

# Detailed search strategies

PubMed (n=4,142)

1."Platelet Aggregation Inhibitors"[Mesh] OR "Platelet Aggregation Inhibitors" [Pharmacological Action] OR "Anticoagulants"[Mesh] OR "Anticoagulants" [Pharmacological Action] OR antiplatelet[tw] OR antithrombotic[tw] OR aspirin[tw] OR acetylsalicylic acid[tw] OR clopidogrel[tw] OR plavix[tw] OR prasugrel[tw] OR ticagrelor[tw] OR brilinta[tw] OR cilostazol[tw] OR ticlopidine[tw] OR cangrelor[tw] OR thienopyridine[tw] OR anticoagulant[tw] OR vitamin K antagonist[tw] OR NOAC[tw] OR NOACs[tw] OR warfarin [tw] OR edoxaban[tw] OR pradaxa[tw] OR dabigatran[tw] OR rivaroxaban[tw] OR xarelto[tw] OR apixaban[tw] OR fondaparinux[tw] OR arixtra[tw] OR ximelagatran[tw] OR vorapaxar[tw] (n=410,008)

2. "Acute Coronary Syndrome"[Mesh] OR "Myocardial Ischemia"[Mesh] OR “Percutaneous Coronary Intervention"[Mesh] OR "Myocardial Revascularization"[Mesh] OR "Myocardial Infarction"[Mesh] OR "Myocardial Reperfusion"[Mesh] OR PCI[tw] OR myocardial infarction[tw] （n=514,171）

3. random* （n=1,342,744）

4. 1 AND 2 AND 3, Filters: Clinical Trial; Publication date from 1995/01/01 to 2020/03/12; Humans; English (n=4,142)

EMBASE (n=7,188)

1. 'Platelet Aggregation Inhibitors' OR anticoagulants OR antiplatelet OR antithrombotic OR aspirin OR acetylsalicylic acid OR clopidogrel OR plavix OR prasugrel OR ticagrelor OR brilinta OR cilostazol OR ticlopidine OR cangrelor OR thienopyridine OR anticoagulant OR ‘vitamin K antagonist’ OR NOAC* OR warfarin OR edoxaban OR pradaxa OR dabigatran OR rivaroxaban OR xarelto OR apixaban OR fondaparinux OR arixtra OR ximelagatran OR vorapaxar (n=486,743)

2. 'Acute Coronary Syndrome' OR 'Myocardial Ischemia' OR 'Percutaneous Coronary Intervention' OR 'Myocardial Revascularization' OR 'Myocardial Infarction' OR 'Myocardial Reperfusion' OR PCI (n=444,248)

3. random* (n=1,955,780)

4. 1 AND 2 AND 3 AND [humans]/lim AND [english]/lim AND [clinical study]/lim AND [1-1-1995]/sd NOT [13-3-2020]/sd (n=7,188)

Cochrane (n=7,019)

1. Platelet Aggregation Inhibitors OR Anticoagulants OR antiplatelet OR antithrombotic OR aspirin OR acetylsalicylic acid OR clopidogrel OR plavix OR prasugrel OR ticagrelor OR brilinta OR cilostazol OR ticlopidine OR cangrelor OR thienopyridine OR anticoagulant OR vitamin K antagonist OR NOAC* OR warfarin OR edoxaban OR Pradaxa OR dabigatran OR rivaroxaban OR xarelto OR apixaban OR fondaparinux OR arixtra OR ximelagatran OR vorapaxar (n=34,871)

2. Acute Coronary Syndrome OR Myocardial Infarction OR Myocardial Ischemia OR Percutaneous Coronary Intervention OR PCI OR Myocardial Revascularization OR Myocardial Reperfusion （n=43,517）

3.random* (n=1,117,505)

4. 1 AND 2 AND 3 with Publication Year from 1995 to 2020, in Trials (n=7,019)

# Table S1. Cochrane risk of bias for the individual studies included.

| **Study name and year** | **Random sequence generation *(Selection bias)*** | **Allocation concealment *(Selection bias)*** | **Blinding of participants and personnel *(Performance bias)*** | **Blinding of outcome assessment *(Detection bias)*** | **Incomplete outcome data *(Attrition bias)*** | **Selective reporting *(Reporting bias)*** | **Other sources of bias** |
| --- | --- | --- | --- | --- | --- | --- | --- |
| **OAC** |  |  |  |  |  |  |  |
| CARS, 1997^[1]^ | 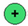 | 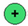 | 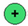 | 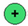 | 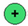 | 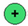 | 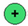 |
| CHAMP, 2002^[2]^ | 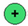 | 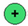 | 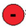§ | 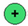 | 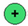 | 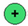 | 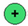 |
| WARIS II, 2002^[3]^ | 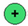 | 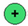 | 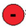§ | 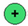 | 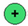 | 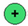 | 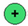 |
| ESTEEM, 2003^[4]^ | 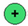 | 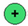 | 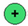 | 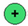 | 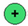 | 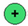 | 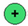 |
| LoWASA, 2004^[5]^ | 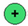 | 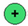 | 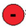§ | 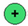 | 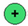 | 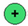 | 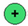 |
| APPRAISE-2, 2011^[6]^ | 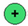 | 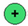 | 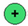 | 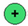 | 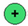 | 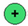 | 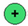 |
| ATLAS ACS 2-TIMI 51, 2012^[7]^ | 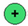 | 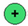 | 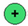 | 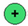 | 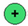 | 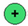 | 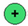 |
| COMPASS, 2017^[8]^ | 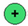 | 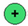 | 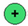 | 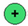 | 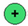 | 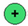 | 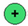 |
| GEMINI-ACS-1, 2017^[9]^ | 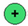 | 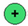 | 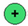 | 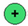 | 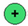 | 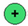 | 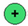 |
| COMMANDER HF, 2018^[10]^ | 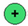 | 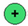 | 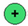 | 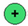 | 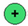 | 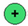 | 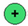 |
| **Antiplatelet** |  |  |  |  |  |  |  |
| OPUS-TIMI 16, 2000^[11]^ | 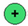 | 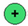 | 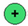 | 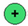 | 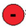† | 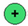 | 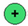 |
| CURE, 2001^[12]^ | 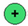 | 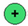 | 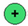 | 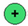 | 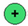 | 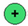 | 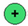 |
| CREDO, 2002^[13]^ | 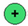 | 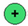 | 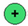 | 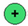 | 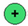 | 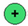 | 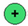 |
| CHARISMA, 2006^[14]^ | 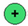 | 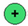 | 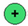 | 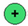 | 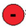† | 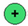 | 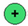 |
| TRITON-TIMI 38, 2007^[15]^ | 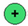 | 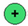 | § |  |  |  |  |
| PLATO, 2009^[16]^ |  |  | § |  | † |  |  |
| Han Y, et al, 2009^[17]^ |  |  |  |  |  |  |  |
| Park SJ, et al, 2010^[18]^ |  |  | § |  |  |  |  |
| TRA2◦P-TIMI 50, 2012^[19]^ |  |  |  |  |  |  |  |
| TRACER, 2012^[20]^ |  |  |  |  |  |  |  |
| EXCELLENT, 2012^[21]^ |  |  | § |  |  |  |  |
| PRODIGY, 2012^[22]^ |  |  | § |  |  |  |  |
| RESET, 2012^[23]^ |  |  | § |  |  |  |  |
| TRILOGY ACS, 2012^[24]^ |  |  | § |  |  |  |  |
| OPTIMIZE, 2013^[25]^ |  |  | § |  |  |  |  |
| DAPT, 2014^[26]^ |  |  |  |  |  |  |  |
| DES LATE, 2014^[27]^ |  |  | § |  |  |  |  |
| SECURITY, 2014^[28]^ |  |  | § |  | † |  |  |
| DAPT, 2015^[29]^ |  |  |  |  |  |  |  |
| ISAR-SAFE, 2015^[30]^ |  |  |  |  |  |  |  |
| PEGASUS-TIMI 54, 2015^[31]^ |  |  |  |  |  |  |  |
| IVUS-XPL, 2016^[32]^ |  |  | § |  |  |  |  |
| OPTIDUAL, 2016^[33]^ |  |  | § |  |  |  |  |
| ITALIC, 2017^[34]^ |  |  | § |  |  |  |  |
| GLOBAL LEADERS, 2018^[35]^ |  |  | § |  | † |  |  |
| SMART-DATE, 2018^[36]^ |  |  | § |  |  |  |  |
| SMART-CHOICE, 2019^[37]^ |  |  | § |  |  |  |  |
| STOPDAPT-2, 2019^[38]^ |  |  | § |  |  |  |  |
| THEMIS, 2019^[39]^ |  |  |  |  | † |  |  |
| TREAT, 2019^[40]^ |  |  | § |  |  |  |  |
| TWILIGHT, 2019^[41]^ |  |  |  |  |  |  |  |
| REDUCE, 2019^[42]^ |  |  | § |  |  |  |  |

= Low risk of bias = High risk of bias

§ Open label non-placebo controlled trials, the study subjects or the investigators were aware of the treatment assignments.

† Not given lost to follow-up information.

# Table S2. Characteristics of included studies

| **Study name and year** | **Clinical setting** | **Inclusion criteria** | **Interventions*** | **Follow-up Duration (months)** | **Sample size*** | **Subgroup of ACS**¶ | **Subgroup of Antiplatelet**¶ |
| --- | --- | --- | --- | --- | --- | --- | --- |
| **OAC** |  |  |  |  |  |  |  |
| CARS, 1997^[1]^ | ACS | 3-21 days after MI in men and postmenopausal or surgically sterilized women | I: Warfarin + ASA,  C: ASA | 14 | I: 5410, C: 5410 | ACS |  |
| CHAMP, 2002^[2]^ | ACS | Patients with MI within the preceding 14 days in veterans | I: Warfarin (Coumadin)+ ASA,  C: ASA alone | 32.4 | I: 2522, C: 2522 | ACS |  |
| WARIS II, 2002^[3]^ | ACS | Patients with MI (a history of typical chest pain; electrocardiographic changes typical of myocardial infarction; and creatine kinase level or aspartate aminotransferase level increase) | I: ①Warfarin+ ASA; ②Warfarin alone,  C: ASA alone | 48.2 | I: 2424, C: 2424 | ACS |  |
| ESTEEM, 2003^[4]^ | ACS | Patients with STEMI or NSTEMI within previous 14 days | I: Ximelagatran,  C: Placebo | 6 | I: 1245, C: 1245 | ACS |  |
| LoWASA, 2004^[5]^ | ACS | Patients with MI within preceding 42 days | I: Warfarin + ASA,  C: ASA alone | 60 | I: 1659, C: 1659 | ACS |  |
| APPRAISE-2, 2011^[6]^ | ACS | Patients with ACS, included UA, NSTE-ACS and STE-ACS | I: Apixaban,  C: Placebo | 8 | I: 3705, C: 3705 | ACS |  |
| ATLAS ACS 2-TIMI 51, 2012^[7]^ | ACS | Hospitalization for ACS within preceding 7 days | I: Rivaroxaban,  C: Placebo | 13.1 | I: 10350, C: 10350 | ACS |  |
| COMPASS, 2017^[8]^ | SCAD | Patients with stable atherosclerotic vascular disease | I: Rivaroxaban + ASA,  C: ASA alone | 23 | I: 9152, C: 9152 | Non-ACS |  |
| GEMINI-ACS-1, 2017^[9]^ | ACS | Patients with ACS, included UA, NSTE-ACS and STE-ACS | I: Rivaroxaban,  C: ASA | 10.9 | I: 1519, C: 1519 | ACS |  |
| COMMANDER HF, 2018^[10]^ | CAD with HF | Patients with CHF>3 months, LVEF<40%, and CAD accompany worsening HF <21 days (atrial fibrillation was excluded in this analysis) | I: Rivaroxaban,  C: Placebo | 21.1 | I: 2507, C: 2507 | Non-ACS |  |
| **Antiplatelet** |  |  |  |  |  |  |  |
| OPUS-TIMI 16, 2000^[11]^ | ACS | Patients with ACS within 72 hours | I: Orbofiban,  C: Placebo | 10 | I: 6867, C: 6867 | ACS | Others |
| CURE, 2001^[12]^ | ACS | Hospitalized within 24 hours after the onset of symptoms and did not have ST -segment elevation | I: DAPT (Clopidogrel +ASA),  C: ASA alone | 9 | I: 6259, C: 6259 | ACS | Long-term vs short-term DAPT |
| CREDO, 2002^[13]^ | PCI | Patients had symptomatic coronary artery disease with objective evidence of ischemia who referred to PCI or thought to be at high risk of PCI | I: DAPT 12 m (Clopidogrel+ ASA),  C: DAPT 1 m (Clopidogrel + ASA) + ASA alone | 12 | I: 1053, C: 1053 |  | Long-term vs short-term DAPT |
| CHARISMA, 2006^[14]^ | CAD or risk factor | Patients showed clinically evident of cardiovascular disease or have multiple risk factors | I: DAPT (Clopidogrel +ASA),  C: ASA alone | 28 | I: 7802, C: 7802 | Non-ACS | Long-term vs short-term DAPT |
| TRITON-TIMI 38, 2007^[15]^ | ACS | Patients with STE-ACS and NSTE-ACS undergoing scheduled PCI with BMS or DES | I: Prasugrel,  C: Clopidogrel | 15 | I: 6813, C: 6813 | ACS | Novel P_2_Y_12_ inhibitor |
| PLATO, 2009^[16]^ | ACS | Patients with NSTE-ACS or STE-ACS during the previous 24 hours | I: Ticagrelor,  C: Clopidogrel | 9.2 | I: 9333, C: 9333 | ACS | Novel P_2_Y_12_ inhibitor |
| Han Y, et al, 2009^[17]^ | ACS | Patients with ACS undergoing PCI with BMS or DES | I: Cilostazol,  C: placebo | 12 | I: 604, C: 604 | ACS | Others |
| Park SJ, et al, 2010^[18]^ | PCI | Patients undergoing PCI with DES at least 12 months ago | I: DAPT (Clopidogrel +ASA),  C: ASA alone | 19.2 | I: 1357, C: 1357 |  | Long-term vs short-term DAPT |
| TRA2◦P-TIMI 50, 2012^[19]^ | ACS | Patients with MI within preceding 2 weeks to 12 months but without scheduled revascularization | I: Vorapaxar,  C: Placebo | 30 | I: 8898, C: 8898 | ACS | Others |
| TRACER, 2012^[20]^ | ACS | Patients with NSTE-ACS | I: Vorapaxar,  C: Placebo | 16.7 | I: 6473, C: 6473 | ACS | Others |
| EXCELLENT, 2012^[21]^ | PCI | Patients undergoing PCI with DES | I: DAPT 12m (Clopidogrel + ASA),  C: DAPT 6m (Clopidogrel + ASA) + ASA alone | 12 | I: 721, C: 721 |  | Long-term vs short-term DAPT |
| PRODIGY, 2012^[22]^ | PCI | Patients undergoing PCI with BMS or DES | I: DAPT 24m (Clopidogrel + ASA),  C: DAPT 6m (Clopidogrel + ASA) + ASA alone | 24 | I: 987, C: 987 |  | Long-term vs short-term DAPT |
| RESET, 2012^[23]^ | PCI | Patients undergoing PCI with DES | I: DAPT 12m (Clopidogrel + ASA),  C: DAPT 3m (Clopidogrel + ASA) + ASA alone | 12 | I: 1058, C: 1058 |  | Long-term vs short-term DAPT |
| TRILOGY ACS, 2012^[24]^ | ACS | Patients with NSTE-ACS without scheduled revascularization | I: Prasugrel,  C: Clopidogrel | 17.1 | I: 4663, C: 4663 | ACS | Novel P_2_Y_12_ inhibitor |
| OPTIMIZE, 2013^[25]^ | PCI | Patients undergoing PCI with DES | I: DAPT 12m (Clopidogrel + ASA),  C: DAPT 3m (Clopidogrel + ASA) + ASA alone | 12 | I: 1556, C: 1556 |  | Long-term vs short-term DAPT |
| DAPT, 2014^[26]^ | PCI | Patients undergoing PCI with DES at least 12 months ago | I: DAPT (Clopidogrel/ Prasugrel + ASA),  C: ASA alone | 33 | I: 5020, C: 5020 |  | Long-term vs short-term DAPT |
| DES LATE, 2014^[27]^ | PCI | Patients undergoing PCI with DES | I: DAPT (Clopidogrel + ASA),  C: ASA alone | 42 | I: 2531, C: 2531 |  | Long-term vs short-term DAPT |
| SECURITY, 2014^[28]^ | PCI | Patients undergoing PCI with DES | I: DAPT 12m (Clopidogrel + ASA),  C: DAPT 6m (Clopidogrel + ASA) + ASA alone | 12 | I: 717, C: 717 |  | Long-term vs short-term DAPT |
| DAPT, 2015^[29]^ | PCI | Patients undergoing PCI with BMS at least 12 months ago | I: DAPT (Clopidogrel/ Prasugrel + ASA),  C: ASA alone | 33 | I: 842, C: 842 |  | Long-term vs short-term DAPT |
| ISAR-SAFE, 2015^[30]^ | PCI | Patients undergoing PCI with DES | I: DAPT 12m (Clopidogrel + ASA),  C: DAPT 6m (Clopidogrel + ASA) + ASA alone | 9 | I: 2003, C: 2003 |  | Long-term vs short-term DAPT |
| PEGASUS-TIMI 54, 2015^[31]^ | ACS | Patients with spontaneous MI 1 to 3 years before enrollment | I: Ticagrelor,  C: Placebo | 33 | I: 14095, C: 14095 | ACS | Long-term vs short-term DAPT |
| IVUS-XPL, 2016^[32]^ | PCI | Patients undergoing PCI with DES (XIENCE PRIME Stent length >45 mm) | I: DAPT 12m (Clopidogrel + ASA),  C: DAPT 6m (Clopidogrel + ASA) + ASA alone | 12 | I: 701, C: 701 |  | Long-term vs short-term DAPT |
| OPTIDUAL, 2016^[33]^ | PCI | Patients with stable CAD or ACS undergoing PCI with DES and have 12 months DAPT after PCI | I: DAPT 48m (Clopidogrel + ASA),  C: DAPT 12m (Clopidogrel + ASA) + ASA alone | 33.4 | I: 695, C: 695 |  | Long-term vs short-term DAPT |
| ITALIC, 2017^[34]^ | PCI | Patients undergoing PCI with DES | I: DAPT 24m (Clopidogrel/ Ticagrelor/ Prasugrel + ASA),  C: DAPT 6m (Clopidogrel/ Ticagrelor/ Prasugrel + ASA) + ASA alone | 24 | I: 924, C: 924 |  | Long-term vs short-term DAPT |
| GLOBAL LEADERS, 2018^[35]^ | PCI | Patients with SCAD or ACS undergone PCI with DES | I: DAPT 12m (Ticagrelor + ASA),  C: DAPT 1m (Ticagrelor + ASA) +Ticagrelor alone | 24 | I: 7988, C: 7988 |  | Long-term vs short-term DAPT |
| SMART-DATE, 2018^[36]^ | PCI | Patients with ACS undergoing PCI with DES | I: DAPT≥12 m (Clopidogrel/ Ticagrelor/ Prasugrel + ASA),  C: DAPT 6 m (Clopidogrel/ Ticagrelor/ Prasugrel + ASA) + ASA alone | 18 | I: 1355, C: 1355 |  | Long-term vs short-term DAPT |
| SMART-CHOICE, 2019^[37]^ | PCI | Patients undergoing PCI with DES | I: DAPT 12m (Clopidogrel/ Ticagrelor/ Prasugrel + ASA),  C: DAPT 3m (Clopidogrel/ Ticagrelor/ Prasugrel + ASA) + P2Y12 inhibitor alone | 12 | I: 1498, C: 1498 |  | Long-term vs short-term DAPT |
| STOPDAPT-2, 2019^[38]^ | PCI | Patients undergoing PCI with DES | I: DAPT 12m (Clopidogrel + ASA),  C: DAPT 1m (Clopidogrel + ASA) + Clopidogrel alone | 12 | I: 1509, C: 1509 |  | Long-term vs short-term DAPT |
| THEMIS, 2019^[39]^ | SCAD | Patients with SCAD and type 2 diabetes mellitus | I: DAPT（Ticagrelor +ASA),  C: ASA alone | 39.9 | I: 9619, C: 9619 | Non-ACS | Long-term vs short-term DAPT |
| TREAT, 2019^[40]^ | ACS | Patients with symptom onset within 24 hours and acute ST-segment elevation on ECG, <75 years of age, and received fibrinolytic therapy | I: Ticagrelor,  C: Clopidogrel | 12 | I: 1913, C: 1913 | ACS | Novel P_2_Y_12_ inhibitor |
| TWILIGHT, 2019^[41]^ | PCI | High-risk patients undergoing PCI with DES | I: DAPT 15m (Ticagrelor + ASA),  C: DAPT 3m (Ticagrelor + ASA) + Ticagrelor alone | 18 | I: 3564, C: 3564 |  | Long-term vs short-term DAPT |
| REDUCE, 2019^[42]^ | ACS | Patients with ACS undergoing PCI with DES | I: DAPT 12 m (Ticagrelor/ Prasugrel/Clopidogrel + ASA),  C: DAPT 3 m (Ticagrelor/ Prasugrel/Clopidogrel + ASA) + ASA alone | 24 | I:745, C: 751 | ACS | Long-term vs short-term DAPT |

**Abbreviations:** OAC: oral anticoagulants; ACS: acute coronary syndrome; CAD: coronary artery disease; SCAD: stable coronary artery disease; HF: heart failure; PCI: percutaneous coronary intervention; MI: myocardial infarction; NSTEMI: Non-ST-elevation myocardial infarction; STEMI: ST-elevation myocardial infarction; UA: unstable angina; NSTE-ACS: non-ST segment elevation acute coronary syndrome; STE-ACS: ST segment elevation acute coronary syndrome; CHF: chronic heart failure; LVEF: left ventricular ejection fraction; DES: drug-eluting stents; BMS: bare-metal stents; DAPT: dual antiplatelet therapy; ASA: aspirin.

* I: intensive antithrombotic therapy; C: conservative antithrombotic therapy.

¶ Details about subgroup analysis of ACS or not and type of antiplatelet.

# Figure S1. Estimates of risk for intracranial hemorrhage between intensive antithrombotic therapy and conservative antithrombotic therapy.

# Figure S2. Estimates of risk for all stroke between intensive antithrombotic therapy and conservative antithrombotic therapy for subgroup of antiplatelet.

# Figure S3. Estimates of risk for ischemic stroke between intensive antithrombotic therapy and conservative antithrombotic therapy for subgroup of antiplatelet.

# Figure S4. Estimates of risk for hemorrhagic stroke between intensive antithrombotic therapy and conservative antithrombotic therapy for subgroup of antiplatelet.

# Figure S5. Estimates of risk for intracranial hemorrhage between intensive antithrombotic therapy and conservative antithrombotic therapy for subgroup of antiplatelet.

# Figure S6. Estimates of risk for all stroke between intensive antithrombotic therapy and conservative antithrombotic therapy for subgroup of ACS.

# Figure S7. Estimates of risk for ischemic stroke between intensive antithrombotic therapy and conservative antithrombotic therapy for subgroup of ACS.

# Figure S8. Estimates of risk for hemorrhagic stroke between intensive antithrombotic therapy and conservative antithrombotic therapy for subgroup of ACS.

# Figure S9. Estimates of risk for intracranial hemorrhage between intensive antithrombotic therapy and conservative antithrombotic therapy for subgroup of ACS.

**Reference**

[1] Fuster V. Randomised double-blind trial of fixed low-dose warfarin with aspirin after myocardial infarction. Lancet 1997;350:389-396.

[2] Fiore LD, Ezekowitz MD, Brophy MT, Lu D, Sacco J, Peduzzi P. Department of Veterans Affairs Cooperative Studies Program Clinical Trial comparing combined warfarin and aspirin with aspirin alone in survivors of acute myocardial infarction: primary results of the CHAMP study. Circulation 2002;105:557‐563.

[3] Hurlen M, Abdelnoor M, Smith P, Erikssen J, Arnesen H. Warfarin, aspirin, or both after myocardial infarction. The New England journal of medicine 2002;347:969-974. Epub 2002/09/27.

[4] Wallentin L, Wilcox RG, Weaver WD, Emanuelsson H, Goodvin A, Nystrom P, Bylock A. Oral ximelagatran for secondary prophylaxis after myocardial infarction: the ESTEEM randomised controlled trial. Lancet 2003;362:789-797. Epub 2003/09/19.

[5] Herlitz J, Holm J, Peterson M, Karlson BW, Evander MH, Erhardt L. Effect of fixed low-dose warfarin added to aspirin in the long term after acute myocardial infarction the LoWASA Study. European Heart Journal 2004;25:232-239.

[6] Alexander JH, Lopes RD, James S, Kilaru R, He Y, Mohan P, Bhatt DL, Goodman S, Verheugt FW, Flather M, et al. Apixaban with antiplatelet therapy after acute coronary syndrome. The New England journal of medicine 2011;365:699-708.

[7] Mega JL, Braunwald E, Wiviott SD, Bassand JP, Bhatt DL, Bode C, Burton P, Cohen M, Cook-Bruns N, Fox KA, et al. Rivaroxaban in patients with a recent acute coronary syndrome. The New England journal of medicine 2012;366:9-19. Epub 2011/11/15.

[8] Eikelboom JW, Connolly SJ, Bosch J, Dagenais GR, Hart RG, Shestakovska O, Diaz R, Alings M, Lonn EM, Anand SS, et al. Rivaroxaban with or without Aspirin in Stable Cardiovascular Disease. The New England journal of medicine 2017;377:1319-1330. Epub 2017/08/29.

[9] Ohman EM, Roe MT, Steg PG, James SK, Povsic TJ, White J, Rockhold F, Plotnikov A, Mundl H, Strony J, et al. Clinically significant bleeding with low-dose rivaroxaban versus aspirin, in addition to P2Y12 inhibition, in acute coronary syndromes (GEMINI-ACS-1): a double-blind, multicentre, randomised trial. Lancet 2017;389:1799-1808.

[10] Zannad F, Anker SD, Byra WM, Cleland JGF, Fu M, Gheorghiade M, Lam CSP, Mehra MR, Neaton JD, Nessel CC, et al. Rivaroxaban in patients with heart failure, sinus rhythm, and coronary disease. New England Journal of Medicine 2018;379:1332-1342.

[11] Cannon CP, McCabe CH, Wilcox RG, Langer A, Caspi A, Berink P, Lopez-Sendon J, Toman J, Charlesworth A, Anders RJ, et al. Oral glycoprotein IIb/IIIa inhibition with orbofiban in patients with unstable coronary syndromes (OPUS-TIMI 16) trial. Circulation 2000;102:149‐156.

[12] Yusuf S, Zhao F, Mehta SR, Chrolavicius S, Tognoni G, Fox KK. Effects of clopidogrel in addition to aspirin in patients with acute coronary syndromes without ST-segment elevation. The New England journal of medicine 2001;345:494‐502.

[13] Steinhubl SR, Berger PB, Mann JT, Fry ET, DeLago A, Wilmer C, Topol EJ. Early and sustained dual oral antiplatelet therapy following percutaneous coronary intervention: a randomized controlled trial. JAMA 2002;288:2411‐2420.

[14] Bhatt DL, Fox KA, Hacke W, Berger PB, Black HR, Boden WE, Cacoub P, Cohen EA, Creager MA, Easton JD, et al. Clopidogrel and aspirin versus aspirin alone for the prevention of atherothrombotic events. The New England journal of medicine 2006;354:1706-1717. Epub 2006/03/15.

[15] Wiviott SD, Braunwald E, McCabe CH, Montalescot G, Ruzyllo W, Gottlieb S, Neumann FJ, Ardissino D, De Servi S, Murphy SA, et al. Prasugrel versus clopidogrel in patients with acute coronary syndromes. The New England journal of medicine 2007;357:2001-2015. Epub 2007/11/06.

[16] Wallentin L, Becker RC, Budaj A, Cannon CP, Emanuelsson H, Held C, Horrow J, Husted S, James S, Katus H, et al. Ticagrelor versus clopidogrel in patients with acute coronary syndromes. The New England journal of medicine 2009;361:1045‐1057.

[17] Han Y, Li Y, Wang S, Jing Q, Wang Z, Wang D, Shu Q, Tang X. Cilostazol in addition to aspirin and clopidogrel improves long-term outcomes after percutaneous coronary intervention in patients with acute coronary syndromes: a randomized, controlled study. Am Heart J 2009;157:733‐739.

[18] Park SJ, Park DW, Kim YH, Kang SJ, Lee SW, Lee CW, Han KH, Park SW, Yun SC, Lee SG, et al. Duration of dual antiplatelet therapy after implantation of drug-eluting stents. The New England journal of medicine 2010;362:1374-1382. Epub 2010/03/17.

[19] Scirica BM, Bonaca MP, Braunwald E, De Ferrari GM, Isaza D, Lewis BS, Mehrhof F, Merlini PA, Murphy SA, Sabatine MS, et al. Vorapaxar for secondary prevention of thrombotic events for patients with previous myocardial infarction: a prespecified subgroup analysis of the TRA 2 degrees P-TIMI 50 trial. Lancet 2012;380:1317-1324. Epub 2012/08/31.

[20] Tricoci P, Huang Z, Held C, Moliterno DJ, Armstrong PW, Van De Werf F, White HD, Aylward PE, Wallentin L, Chen E, et al. Thrombin-receptor antagonist vorapaxar in acute coronary syndromes. The New England journal of medicine 2012;366:20-33.

[21] Gwon HC, Hahn JY, Park KW, Song YB, Chae IH, Lim DS, Han KR, Choi JH, Choi SH, Kang HJ, et al. Six-month versus 12-month dual antiplatelet therapy after implantation of drug-eluting stents: the Efficacy of Xience/Promus Versus Cypher to Reduce Late Loss After Stenting (EXCELLENT) randomized, multicenter study. Circulation 2012;125:505‐513.

[22] Valgimigli M, Campo G, Monti M, Vranckx P, Percoco G, Tumscitz C, Castriota F, Colombo F, Tebaldi M, Fucà G, et al. Short- versus long-term duration of dual-antiplatelet therapy after coronary stenting: a randomized multicenter trial. Circulation 2012;125:2015‐2026.

[23] Kim BK, Hong MK, Shin DH, Nam CM, Kim JS, Ko YG, Choi D, Kang TS, Park BE, Kang WC, et al. A new strategy for discontinuation of dual antiplatelet therapy: the RESET Trial (REal Safety and Efficacy of 3-month dual antiplatelet Therapy following Endeavor zotarolimus-eluting stent implantation). J Am Coll Cardiol 2012;60:1340-1348. Epub 2012/09/25.

[24] Roe MT, Armstrong PW, Fox KA, White HD, Prabhakaran D, Goodman SG, Cornel JH, Bhatt DL, Clemmensen P, Martinez F, et al. Prasugrel versus clopidogrel for acute coronary syndromes without revascularization. The New England journal of medicine 2012;367:1297‐1309.

[25] Feres F, Costa RA, Abizaid A, Leon MB, Marin-Neto JA, Botelho RV, King SB, Negoita M, Liu M, de Paula JE, et al. Three vs twelve months of dual antiplatelet therapy after zotarolimus-eluting stents: the OPTIMIZE randomized trial. JAMA 2013;310:2510‐2522.

[26] Mauri L, Kereiakes DJ, Yeh RW, Driscoll-Shempp P, Cutlip DE, Steg PG, Normand SL, Braunwald E, Wiviott SD, Cohen DJ, et al. Twelve or 30 months of dual antiplatelet therapy after drug-eluting stents. The New England journal of medicine 2014;371:2155-2166. Epub 2014/11/18.

[27] Lee CW, Ahn JM, Park DW, Kang SJ, Lee SW, Kim YH, Park SW, Han S, Lee SG, Seong IW, et al. Optimal duration of dual antiplatelet therapy after drug-eluting stent implantation: a randomized, controlled trial. Circulation 2014;129:304‐312.

[28] Colombo A, Chieffo A, Frasheri A, Garbo R, Masotti-Centol M, Salvatella N, Oteo Dominguez JF, Steffanon L, Tarantini G, Presbitero P, et al. Second-generation drug-eluting stent implantation followed by 6- versus 12-month dual antiplatelet therapy: the SECURITY randomized clinical trial. JACC 2014;64:2086‐2097.

[29] Kereiakes DJ, Yeh RW, Massaro JM, Driscoll-Shempp P, Cutlip DE, Steg PG, Gershlick AH, Darius H, Meredith IT, Ormiston J, et al. Antiplatelet therapy duration following bare metal or drug-eluting coronary stents: the dual antiplatelet therapy randomized clinical trial. JAMA 2015;313:1113‐1121.

[30] Schulz-Schupke S, Byrne RA, Ten Berg JM, Neumann FJ, Han Y, Adriaenssens T, Tolg R, Seyfarth M, Maeng M, Zrenner B, et al. ISAR-SAFE: a randomized, double-blind, placebo-controlled trial of 6 vs. 12 months of clopidogrel therapy after drug-eluting stenting. European heart journal 2015;36:1252‐1263.

[31] Bonaca MP, Bhatt DL, Cohen M, Steg PG, Storey RF, Jensen EC, Magnani G, Bansilal S, Fish MP, Im K, et al. Long-term use of ticagrelor in patients with prior myocardial infarction. The New England journal of medicine 2015;372:1791‐1800.

[32] Hong SJ, Shin DH, Kim JS, Kim BK, Ko YG, Choi D, Her AY, Kim YH, Jang Y, Hong MK. 6-Month Versus 12-Month Dual-Antiplatelet Therapy Following Long Everolimus-Eluting Stent Implantation: the IVUS-XPL Randomized Clinical Trial. JACC Cardiovascular interventions 2016;9:1438‐1446.

[33] Helft G, Steg PG, Le Feuvre C, Georges JL, Carrie D, Dreyfus X, Furber A, Leclercq F, Eltchaninoff H, Falquier JF, et al. Stopping or continuing clopidogrel 12 months after drug-eluting stent placement: the OPTIDUAL randomized trial. European heart journal 2016;37:365‐374.

[34] Didier R, Morice MC, Barragan P, Noryani AAL, Noor HA, Majwal T, Hovasse T, Castellant P, Schneeberger M, Maillard L, et al. 6- Versus 24-Month Dual Antiplatelet Therapy After Implantation of Drug-Eluting Stents in Patients Nonresistant to Aspirin: Final Results of the ITALIC Trial (Is There a Life for DES After Discontinuation of Clopidogrel). JACC Cardiovasc Interv 2017;10:1202-1210. Epub 2017/06/24.

[35] Vranckx P, Valgimigli M, Jüni P, Hamm C, Steg PG, Heg D, van Es GA, McFadden EP, Onuma Y, van Meijeren C, et al. Ticagrelor plus aspirin for 1 month, followed by ticagrelor monotherapy for 23 months vs aspirin plus clopidogrel or ticagrelor for 12 months, followed by aspirin monotherapy for 12 months after implantation of a drug-eluting stent: a multicentre, open-label, randomised superiority trial. Lancet (london, england) 2018;392:940‐949.

[36] Hahn JY, Song YB, Oh JH, Cho DK, Lee JB, Doh JH, Kim SH, Jeong JO, Bae JH, Kim BO, et al. 6-month versus 12-month or longer dual antiplatelet therapy after percutaneous coronary intervention in patients with acute coronary syndrome (SMART-DATE): a randomised, open-label, non-inferiority trial. J Thromb Haemost 2018;391:1274-1284. Epub 2018/02/01.

[37] Hahn JY, Song YB, Oh JH, Chun WJ, Park YH, Jang WJ, Im ES, Jeong JO, Cho BR, Oh SK, et al. Effect of P2Y12 Inhibitor Monotherapy vs Dual Antiplatelet Therapy on Cardiovascular Events in Patients Undergoing Percutaneous Coronary Intervention: The SMART-CHOICE Randomized Clinical Trial. JAMA 2019;321:2428-2437. Epub 2019/06/27.

[38] Watanabe H, Domei T, Morimoto T, Natsuaki M, Shiomi H, Toyota T, Ohya M, Suwa S, Takagi K, Nanasato M, et al. Effect of 1-Month Dual Antiplatelet Therapy Followed by Clopidogrel vs 12-Month Dual Antiplatelet Therapy on Cardiovascular and Bleeding Events in Patients Receiving PCI: The STOPDAPT-2 Randomized Clinical Trial. JAMA 2019;321:2414-2427. Epub 2019/06/27.

[39] Steg PG, Bhatt DL, Simon T, Fox K, Mehta SR, Harrington RA, Held C, Andersson M, Himmelmann A, Ridderstråle W, et al. Ticagrelor in Patients with Stable Coronary Disease and Diabetes. The New England journal of medicine 2019;381:1309-1320. Epub 09/01.

[40] Berwanger O, Lopes RD, Moia DDF, Fonseca FA, Jiang L, Goodman SG, Nicholls SJ, Parkhomenko A, Averkov O, Tajer C, et al. Ticagrelor Versus Clopidogrel in Patients With STEMI Treated With Fibrinolysis: TREAT Trial. JACC 2019;73:2819‐2828.

[41] Mehran R, Baber U, Sharma SK, Cohen DJ, Angiolillo DJ, Briguori C, Cha JY, Collier T, Dangas G, Dudek D, et al. Ticagrelor with or without Aspirin in High-Risk Patients after PCI. The New England journal of medicine 2019;381:2032-2042. Epub 09/26.

[42] De Luca G, Damen SA, Camaro C, Benit E, Verdoia M, Rasoul S, Liew HB, Polad J, Ahmad WA, Zambahari R, et al. Final results of the randomised evaluation of short-term dual antiplatelet therapy in patients with acute coronary syndrome treated with a new-generation stent (REDUCE trial). EuroIntervention : journal of EuroPCR in collaboration with the Working Group on Interventional Cardiology of the European Society of Cardiology 2019;15:e990-e998.
